# Retinal Encoding of Ultrabrief Shape Recognition Cues

**DOI:** 10.1371/journal.pone.0000871

**Published:** 2007-09-12

**Authors:** Ernest Greene

**Affiliations:** 1 Laboratory for Neurometric Research, Department of Psychology, University of Southern California, Los Angeles, California, United States of America; 2 Neuropsychology Foundation, Sun Valley, California, United States of America; The Rockefeller University, United States of America

## Abstract

Shape encoding mechanisms can be probed by the sequential brief display of dots that mark the boundary of the shape, and delays of less that a millisecond between successive dots can impair recognition. It is not entirely clear whether this is accomplished by preserving stimulus timing in the signal being sent to the brain, or calls for a retinal binding mechanism. Two experiments manipulated the degree of simultaneity among and within dot pairs, requiring also that the pair members be in the same half of the visual field or on opposite halves, *i.e.*, across the midline from one another. Recognition performance was impaired the same for these two conditions. The results make it likely that simultaneity of cues is being registered within the retina. A potential mechanism is suggested, calling for linkage of stimulated sites through activation of PA1 cells. A third experiment confirmed a prior finding that the overall level of recognition deficit is partly a function of display-set size, and affirmed submillisecond resolution in binding dot pairs into effective shape-recognition cues.

## Introduction

*“Certainly every Gestalt psychologists knows perfectly well that a beam of light falls on a certain retinal spot as if there were no other retinal points and no other light beams upon them. But there is no proof that the physiological sequel of such stimulation is made up of local independent processes.”* Kohler [1]

Previous research has shown that objects can be identified when they are represented using a relatively sparse sampling of dots that mark the outer boundary, similar to a silhouette [2]. Further, they can be recognized with each dot being shown sequentially for only a tenth of a millisecond [3], [4], which can be described as a minimal transient discrete cue (MTDC) protocol for the study of shape perception.

To be effective as shape-recognition cues, the dots also must be presented with millisecond-levels of temporal contiguity. If adjacent dots are displayed in pairs, providing delays between successive pairs as small as 2 ms impairs recognition, as do delays as short as 0.5 ms between the pair members [5], [6].

Such very brief time intervals might call for a special retinal encoding mechanism that results in synchronous firing of retinal ganglion cells. A number of investigators have proposed that synchrony serves to bind figural components [7]–[12], or to offset redundant information loss [13].

There is evidence that simultaneity is preserved in the signal being sent to the brain. Conduction velocity of optic nerve fibers appears to be designed to preserve the time differentials among stimulus events, or lack thereof. Stanford [14] reported that travel time, which was largely a function of axon diameter, was faster for signals coming from more peripheral portions of the retina. This provided for equal time-of-arrival at the lateral geniculate nucleus irrespective of the retinal site that was stimulated. The travel time for arrival at the thalamus was in the range of 5 ms, with a standard deviation of approximately 0.5 ms. Reinagel & Reid [15], [16] report that the signals being received by the lateral geniculate nucleus are reliable, temporally precise, and are altered very little by noise.

However, even if the retina provides special mechanisms for registering the simultaneity of stimulation with high precision, this might mediate motion analysis and have no relevance to shape recognition. In general one would not expect any coordination of activity within the retina whose purpose was to modify the utility of shape-recognition cues. The signal may be filtered for contrast, motion and other local properties, but it is thought that global relationships are evaluated at a very late stage – perhaps in the lateral occipital complex, which is a major brain site for analysis of shape information in humans [17].

**Figure 1:**
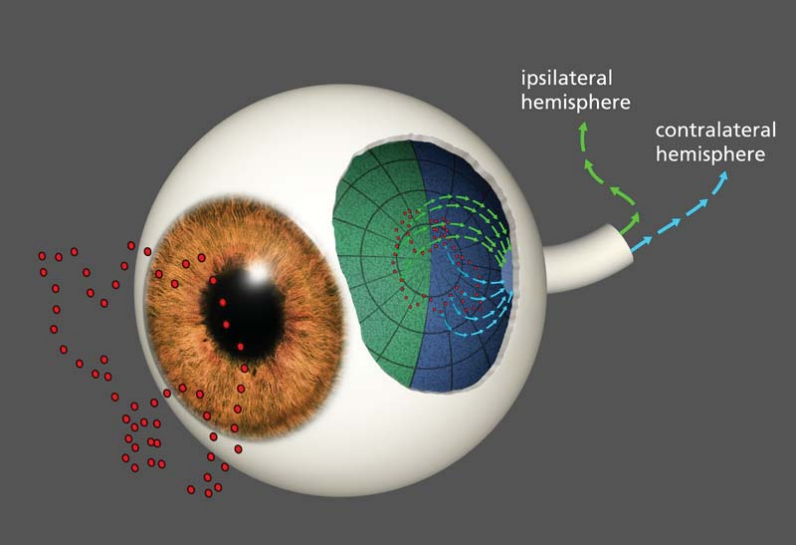
Stimulus dots on each side of the display go to opposite hemispheres. The vertical meridian of the eye provides a dividing line for both anatomical and functional segregation of image information. As subjects fixate the display board, dots that are on each side of the stimulus will be sent to opposite hemispheres. This is true even when both eyes are used to view the stimulus, as illustrated in any basic textbook on visual function. Thus for dot pairs that are horizontally aligned, one member will be sent to the ipsilateral side of the brain and the other to the contralateral side. This might be expected to impair performance in a minimal transient discrete cue task wherein pairs must have millisecond-level contiguity to provide for effective shape recognition.

Further, even if the retina can transmit the timing with great precision, this might provide functional benefit only for messages coming from each half of the retina. As illustrated in Figure 1, nasal and temporal hemiretinas send their information to different hemispheres. Even with binocular viewing, the projections of the optic nerves carry information from the right visual field, *i.e*., the right half of the display board in the present case, to the left hemisphere, and vice versa. These signal streams will be processed through several neural links, including a requirement for the signal to cross the corpus callosum, before relationships between the two sides of the image can be assessed by the brain. It seems unlikely that millisecond-level precision can be preserved across so many processing stages, and if not, one would find that linkage of contemporaneous shape cues would be restricted to a given hemiretina. If this is the case, one would predict that any benefit from contemporaneous stimulation would be manifested only within each half of the visual field, *i.e.*, for image components that are provided to the left half or to the right half of the display.

Two experiments are reported that provide clear evidence that the two halves of the visual field are linked by a mechanism that registers contemporaneous shape cues. This is best explained on the basis of local encoding mechanisms that link the shape cues, as discussed below.

A third experiment was performed to answer some questions about the mechanism itself. As noted above, two earlier reports [4], [5] found significant declines in recognition of shapes when near simultaneous dot-pairs were temporally separated by 2 ms. Further, with the pairs being separated by 3 ms, inserting a half millisecond interval between the pair members produced a significant decline in recognition. It is possible that a requirement for sub-millisecond simultaneity is a special requirement for individual dots, providing some form of linkage that makes them more effective at eliciting shape memory. It cannot be assumed that any additional linkage would be seen with dot pairs in which the members have already been yoked. Further, temporal proximity effects for intervals shorter than 2 ms appeared to be stronger than for intervals greater than 2 ms, which again might indicate two separate mechanisms – the former for individual dots and the latter for pairs in which the individual dots have already been linked. One goal of the third experiment was to clarify this matter by providing submillisecond separation of successive dot-pairs.

One of the previous studies also observed that the temporal proximity had a differential impact on recognition as a function of the number of dots that were used as shape cues [6]. An early goal in the development of the research protocol was to compensate for complexity and familiarity of shapes by varying the size of the display set, thus providing for approximate equality in potential for recognition. However, if it could be confirmed that recognition is a function of the number of dots in the display set, this might provide an additional tool for examining the mechanism by which the shape cues are integrated.

The third experiment therefore examined recognition of shapes with brief display of pairs of boundary dots, evaluating with temporal separation between successive pairs at intervals ranging from 0.5 to 8 ms, and with attention to differential effects as a function of the number of dots in the display set. The results indicate linear gradients of effect across time and display set size.

## Results

### Experiment 1: Differential display of dot pairs to retinal hemifields

**Figure 2:**
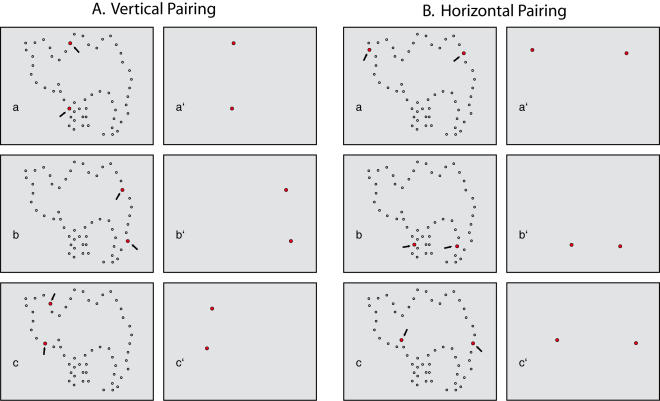
Horizontal or vertically alignment of pairs. The four panels illustrate the method of forming vertically and horizontally aligned pairs. In the first and third panels a full display set is shown, these being all the dots that were paired and successively displayed to a given participant. To form the pair a dot was chosen at random, and then was yoked with another, requiring that the axis of alignment across the two must be approximately vertical or horizontal. This process was repeated until all possible pairs were chosen. The second and fourth panels show the pairs being successively displayed.

As illustrated in Figure 2, each shape was displayed as a spaced sampling of dots that fell on the outer boundary of the shape, similar to a silhouette. The dots of this display set were flashed successively on a 64×64 LED array, with the duration of each dot being 0.1 ms (designated as T1). Dots of the display set were shown in pairs that were chosen to have an axis of alignment that was predominantly vertical or horizontal, as illustrated in the left panels of Figure 2A and 2B, respectively. Except for the constraint on alignment, the locations of pair members were chosen at random.

There was no delay in display of a given pair, which is to say that the T2 interval was zero. Thus as soon as the first dot was turned off, the second was turned on, and total display time for the pair was 0.2 ms. The interval between successive, randomly chosen pairs, designated as T3, was varied from 0–6 ms. The right panels of Figure 1A and 1B show each pair in isolation, suggesting what would stimulate the retina in the 0.2 ms required to show the pair, and with successive pairs being separated by a T3 interval.

An inventory of 150 shapes was shown to each participant, each shape being shown only once using a particular combination of treatment conditions, *i.e.*, vertical or horizontal alignment of pairs, and one of the four T3 intervals. The participants were asked to name each shape, and their answers were scored as indicating recognition or not. In the analysis that follows, level of recognition may also be identified as hit rate.

**Figure 3:**
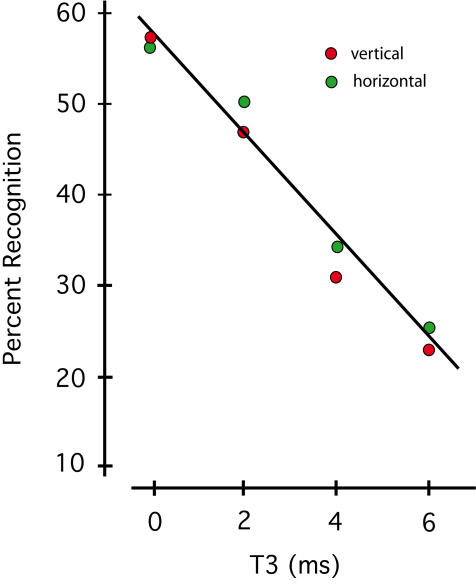
Lack of horizontal/vertical differential indicates retina cue-binding. Experiment 1 examined dot-pair alignment and temporal separation of pairs as factors for successful recognition of the shapes. Each dot pair was shown in 0.2 ms, and the interval between successive pairs was varied from 0–6 ms. Model values reflecting mean recognition level for each of the eight treatment combinations have been plotted. There was no indication of a difference in recognition as a function of vertical or horizontal dot alignment, but there was a significant linear decline as a function of the temporal interval between dot pairs.

The data were evaluated with a generalized linear mixed model (GLMM), which found a significant decline in recognition as a function of T3 interval (p<.001). There were no significant differences for vertical versus horizontal alignment of the dot pairs (p = .50), no interaction between alignment and T3 (p = .51) and no sign of departure from linearity across the four levels of T3 (p = .54). Model means are plotted in Figure 3.

Since dot alignment was not significant, a single regression line has been fitted to the average of the two conditions. A linear decline in recognition is clearly evident, with the differential between 0 ms and 2 ms being significant (p<.01), which agrees with the results of previous work [5].

Finding that pair members are equally effective as shape cues whether they have horizontal or vertical alignment provides clear evidence of functional linkage across the midline of the visual field. The prior work [5], [6] has shown that the members of the pair must be presented with near simultaneity to be maximally effective as shape cues, and temporal separations of even half a millisecond can produce a significant impairment of recognition. With each pair being displayed for only 0.2 ms, both members of a vertically aligned pair would fall on either the nasal hemiretina or temporal hemiretina irrespective of where the subject was looking. Conversely, the horizontal positioning of pair members would place each member on opposite sides of the vertical meridian. One would have seen a differential in the level of recognition if the members of the pair could not be functionally linked with half-millisecond precision. The fact that no differential in recognition was found for the horizontal- and vertical-pairing conditions indicates that the pair members were provided with that linkage.

To be completely accurate, if the participant was not fixating on the center of the display – even though central fixation would be optimal for performing the recognition task – a few horizontally aligned pairs would likely stimulate the same side of the retina. However, even here most pair members would fall on opposite sides, and one would see dramatic impairment of recognition if the two sides were not functionally linked. This is best explained in terms of a functional linkage of the two hemiretinas, as discussed below.

### Experiment 2: Retinal hemifields register differentials in the millisecond range

Previous studies using the MTDC protocol [5], [6] found the strongest evidence of the importance of stimulus simultaneity by keeping a constant T3 interval between pairs and varying the T2 interval. Experiment 2 followed that procedure, setting T3 at 3 ms and using four intermediate levels of T2. Only horizontal pairs were used to focus the full effort on an examination of temporal linkage of hemiretinas.

GLMM analysis found a significant decline in recognition as a function of the T2 interval (p<.001). The differential between 0.0 and 1.0 ms was significant (p<.02), which is consistent with earlier findings that millisecond levels of delay between pair members will impair recognition [5].

**Figure 4:**
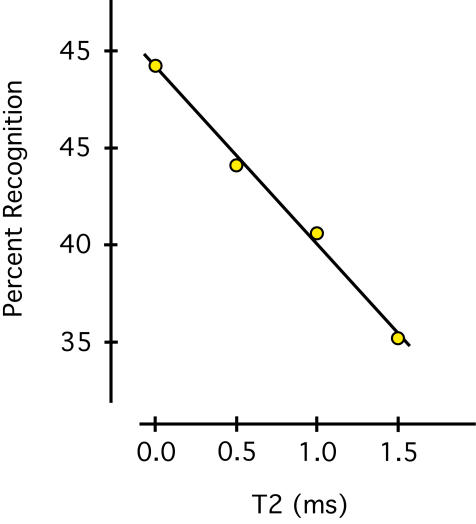
Manipulation of between-pair timing provides similar evidence. Experiment 2 used horizontally aligned dots, kept the interval between successive pairs at a constant 3 ms, and varied the interval between members of each pair from 0–1.5 ms. There was a significant linear decline in recognition as the interval between pair members was increased.

Predicted means are plotted in Figure 4, along with a regression line that affirms the linear decline in treatment effect. The decline demonstrates that the pair members are most effective as shape-recognition cues if they are contemporaneous. This further supports the inference that the two sides of the retina are functionally linked.

### Experiment 3: Submillisecond dot-pair effects and role of display set size

The third experiment examined whether the slope of the T3 timing differentials might change as one transitioned into the submillisecond range, and evaluated whether the number of dots in the display set affected the level of recognition. Display set size, *i.e*., the number of dots being used to display a given shape, may also be designated as Dot#. To provide maximal comparability to prior work [5], [6], pair membership was randomly selected.

The GLMM analysis found significant declines in recognition with increases in T3 interval or Dot# (p<.001 for each). There was no indication of nonlinear components for T3 (p = 0.18) or for Dot# (p = 0.61). There was a significant interaction between T3 and Dot# (p = .02), with the impact on recognition becoming greater with increases in Dot# and T3 interval.

**Figure 5:**
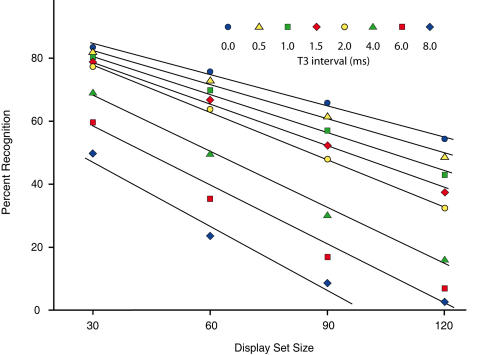
Decline of recognition for T3 intervals as a function of Dot#. For Experiment 3, model predictions at the eight T3 intervals are plotted across the four display set sizes that were chosen for analysis. Recognition declined in a systematic manner as the number of dots in the display set was increased, with very consistent alignment of plot points except for the largest display set shown at the longest T3 interval.

**Figure 6:**
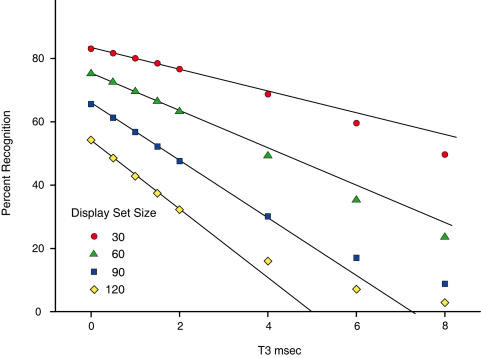
Decline of recognition for Dot# groups as a function of T3 interval. The model predictions for the four chosen display set sizes of Experiment 3 are plotted across the range of T3 intervals. Recognition declined in a systematic manner as a function of the delay between successive dot pairs. Alignment across plot points was most consistent for T3 intervals in the 0–2 ms range.

Recognition levels derived from the GLMM model were backtransformed, and the predictions at 30, 60, 90 and 120 dots were used to plot treatment effects. Figures 5 and 6 show these values, with display set size (Dot#) being specified on the abscissas in the first figure, and T3 in the second. Trend lines have been added to emphasize the most consistently aligned points.

Figure 5 plots show consist differentials for the T3 intervals when each is plotted against the Dot# levels. The plot points for T3 intervals of 2 ms or less were the most consistently aligned, and the trend lines have been fitted using only these points. Plot points for intervals greater than 2 ms are also reasonably well aligned. The worst alignment can be seen for T3 equal to 8 ms where there were 120 dots in the display set, but it is doubtful that the recognition level is reliably greater than zero, which is the prediction of the trend line.

In Figure 6 one can see that for each of the four levels of Dot#, there was a consistent decline of recognition as a function of the T3 interval, and the differentials in display set size yielded reliable differences in recognition level. Two points deviated from the suggested trend lines, these being for the 120-dot set at T3 intervals of 6 and 8 ms. As for the discrepancy discussed for Figure 5, these values are close to the linear prediction of zero recognition.

One might note that the 30- and 60-dot plot points in Figure 6 tend to fall at lower levels of recognition than would be predicted by the trend lines, and for 90- and 120-dot sets the plot points are higher that predicted. If these prove to be reliable deviations from linearity, they would essentially cancel out when combining across the full inventory of shapes, thus yielding a linear decline for the overall mean from 0 to 8 ms, as was found in earlier work [5]. This suggests that the use of mean hit-rate may not properly estimate declines at long intervals, and one should be careful in accepting the simple linear plots as a reflection of neural mechanism.

In both figures (5 and 6) one can see the interaction of T3 with Dot# as a progressive spread of plot points (and trend lines) as one moves from left to right – possibly more so in the 0–2 ms range. This provides visual confirmation of the statistically significant interaction reported above.

The spacing factor had been chosen during early development of research protocols, having the goal of providing shapes that were approximately equivalent in their potential for recognition. Recognition levels had been tested with both T2 and T3 set to zero, and with the dots being delivered in the order given in the original address list. This provided for hit rates in the 70% range for each of the shapes. At least two subsequent experiments failed to note any differential as a function of display set size, the one closest the present work [5] finding that recognition level for T2 and T3 = 0 was approximately level across the full range of Dot#.

The present results indicate that the adjustment for display set size did not render the shapes comparable in their potential for recognition, in that hit rates were lower as a function of Dot# for each level of T3, including T3 = 0. It is unclear whether this discrepancy should be attributed to a different sampling of test subjects. Whatever the case, it provides an additional caution against relying too heavily on any given experiment, even where the significance levels are suitably high.

It should be clear that T3 and Dot# both contribute to the total time required to display the shapes, and total time for the four levels of Dot# were as follows, with the size of the display set shown in parentheses: (30) 3–123 ms; (60) 6–246 ms; (90) 9–369 ms; (120) 12–492 ms

If impairment of recognition is a function of total display time, then a plot of one against the other should provide a single function for each combination of Dot# and T3 interval. An evaluation of just the 2–8 ms (T3) range seemed to suggest such a function, meaning that one could predict recognition level using only the total time of display, irrespective of how many dots were in the display set. However, for data from T3 = 0–2 ms there were clear and consistent differences in recognition for the four levels of display-set size. Further, if total time for display was plotted for all levels of T3, with Dot# levels being designated by specific symbols, there was sufficient alignment within each Dot# subset that one could make the case that recognition was not solely a function of total display time. There was some indication that the plots converged to zero somewhere near 275 ms, but this could not be affirmed with any certainty given the lack of correspondence of the display time across the four subsets. So while it is possible that recognition deficits are due to the total time required to display the dots if one is using a T3 of 2 ms or longer, even that cannot be affirmed with any confidence. At this point it would be best to await more definitive results.

## Discussion

The third experiment provides data that clarifies and extends earlier findings, and will be discussed first. The prior work found a linear decline in recognition, assessed for the average across all shapes, and measured at T3 intervals of 2 ms and longer [5.6]. The present work extends the finding to T3 intervals shorter than 2 ms, and finds no clear evidence for a change in slope across the two time ranges. This does not rule out the possibility that the strength of effect might differ for dot subsets having more or less than two dots.

The number of dots in the display set (Dot#) produced major differentials in shape recognition. Each of the four levels of Dot# that was chosen for evaluation, *i.e*., 30, 60, 90 and 120 dots, manifested a linear decline across the 0–2 ms range of T3, with hit-rates for the 2–8 ms range being progressively less linear for each level of Dot# that was examined. In like manner, recognition performance for each of the T3 treatments declined in a linear manner across the four levels of Dot# that were chosen for analysis. While one might think this to be a foregone conclusion given that the plots represent two views of the same experimental results, it would be entirely possible for Dot# to manifest nonlinear effects while T3 intervals were showing linear declines.

The results indicate that recognition performance is determined by the degree of simultaneity of the dot display. With a T3 interval of zero, all dots in the display set are delivered as quickly as possible given the design of the display board, this being 0.1 ms multiplied by the number of dots in the display set. Thus for the 30-dot display set the entire complement is shown in 3 ms, and for the 120-dot set the shape is delivered in 12 ms. By most standards of work on object/shape recognition, the time required to deliver the full display set is very short, even for shapes having a large set size. But although only 12 ms was needed to show a shape using 120 dots, there was a significant decrement of recognition, which suggests that optimal recognition should require that all the dots be presented in far less time, perhaps at the same moment.

A standard model for the integration of information over time delays explains the process as one in which briefly displayed information is labile, meaning that it persists only for a limited amount of time. The stimulus is considered to be most salient when it is first displayed, but as the moments pass it becomes less salient and thus less effective as a cue. If one presents two or more brief stimuli in succession, the ones that were shown at an earlier point will have suffered more decay, and the aggregate set will have a net salience that reflects the overall amount of decay among the cues. For reviews of the persistence concept, see [18]–[20]. For additional background on the role of information persistence in shape recognition, see [3].

The information persistence model does not seem appropriate for explaining the current results. Virtually all the data to date using the MTDC protocol has indicated linear declines in recognition with changes in timing and/or number of dots in the display set. Biological activities generally do not manifest linear decay curves, and such declines are more commonly found with interruption of an iterative, information processing mechanism. Even if one assumes that a linear decay is possible, it would have to be specific to a given range of T3 and Dot#. If persistence is short, allowing for a linear decline with small display sets and short T3 intervals, there should be too few salient dots available for recognition with large display sets and at long T3 intervals. Conversely, if persistence is long enough to allow a linear prediction for the large sets and long display times, one would not have simple linear declines of recognition for small display sets at short T3 intervals.

The concept of stimulus binding faces similar problems if simultaneity acts to increase the salience of shape cues by binding successive pairs. Here one would not expect any differentials as a function of the number of pairs, and total display time should be irrelevant. The binding would have to extend downstream to far distant pairs, and it is unclear how it would do so in a linear manner irrespective of the time differentials being provided. For more on the binding hypothesis, see [7]–[12].

Turning to the locus of effect, the first two experiments provided evidence that the requirement for millisecond-level simultaneity was the same whether the two members of a pair were presented to the same hemiretina, *i.e.*, in the same half of the visual field, or were shown with one member falling on the nasal hemiretina and the other on the temporal hemiretina.

As outlined in the Introduction, in theory one might avoid the conclusion of retinal linkage of contemporaneous stimuli if the timing of the stimulus events were being preserved in the signal being sent to higher brain centers. There is some evidence for such millisecond-level precision, in that a number of laboratories have reported highly correlated firing in retinal ganglion cells [21]–[34]. There are, however, reasons to be cautious in declaring that millisecond-level differentials in stimulus timing are being preserved in the retinal signal. Most of the evidence supporting millisecond levels of precision has been based on correlations of spontaneous activity, electrically stimulated activity, or averaging across extended periods of time. When brief light flashes are used, response to initial onset of the stimulus is commonly found to be quite variable. Murphey & Rieke [35], for example, measured responses in three kinds of mouse ganglion cells with stimuli that were reasonably well restricted to the excitatory receptive field of the cells. They found that the standard deviation to first-spike response varied from 2–10 ms. Maunsell *et al*. [36] also found substantial variability of response to stimulus onset in the signal being received in lateral geniculate nucleus of *Macaque*. At the very least one should allow that highly precise encoding of stimulus timing is not a general property of retinal signals.

Further, whether or not the optic nerve signal can preserve the timing of very brief stimulation, it is unlikely that the present results can be explained in terms of high precision signal transmission. Where the dot pairs were horizontally aligned, one fell on the nasal hemiretina and the other on the temporal hemiretina, and the signals that were generated were sent to different hemispheres. Even if one posits temporal precision in the millisecond range up to V1, it is unlikely that it could be preserved on through the corpus callosum, as required for linking a cue in one visual field with a contemporaneous cue in the other visual field. Finding that horizontal pairs as well as vertical pairs are equally sensitive to simultaneity of display argues that the linking mechanism is in the retina.

Polyaxonal amacrine (PA1) cells, first characterized in primate retina [37], have anatomical features that may be suitable for registering and coordinating among spatially and temporally discrete stimulus events. Across a number of species, these cells have relatively narrow dendritic fields, but also have axons with a prolific branching pattern that extends over a much larger area [37]–[43]. In *Macaque* the diameters of the dendritic fields measure about 200 microns in central vision and 600 microns in the periphery, with the span of the axon field being roughly 10 times larger [37]. If similar dimensions can be assumed for the PA1 cells of human retina, we can use the unit conversions reported by Dacey [44] to estimate diameters of the axonal arbors to be about 7.5 degrees of visual angle for central vision, and 40 degrees of visual angle in the periphery. Wright and Vaney [45] report that PA1 cells appear to connect to a single class of ganglion cell, which may be “local edge detectors.”

**Figure 7:**
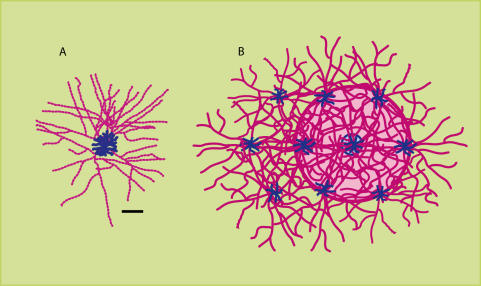
PA1 cells may register contemporaneous stimulus events. The polyaxonal amacrine cell may provide a mechanism for linking discrete stimulus events. A. This illustration approximates the camera Lucida drawing by Dacey [37] of a typical primate polyaxonal amacrine cell. The dendritic branches are shown in blue, and evidence indicates that stimulus influence is restricted to this zone. The axon branches are shown in red. These branches have vericosities along their full length that would provide a spreading ring of synaptic influence upon activation of the cell. The scale bar is 500 microns. B. An array of polyaxonal cells is provided to illustrate linkage of cells through an extensive overlapping axon plexus. The span of the axon field, shown in pink, ranges from roughly 7.5 to 40 degrees of visual angle for central and peripheral retina, respectively. For any given location of the retina, axons from approximately 200–300 PA1 cells overlap, providing a plexus with an average spacing of approximately 2 microns [45].

Figure 7A illustrates a typical PA1 cell, showing a relatively small dendritic field and a far larger axonal arbor. It is unlikely that the cell responds to stimuli that are much bigger than its dendritic field. Most recordings from axon-bearing amacrine cells indicate that the receptive fields are co-extensive with the dendritic fields or only slightly larger [39], [43], [45]–[47]. The dendritic trees are relatively compact, which would be optimal for registering stimulation by discrete boundary markers, *e.g.*, dots. But once activated, the extended axonal plexus could serve to link contemporaneous events being generated at two or more locations [37], [45].

Figure 7B illustrates an array of PA1 cells, showing the ability to distribute activity from the localized stimulation over large spans. It should be noted that the actual cell density is far greater than suggested in the illustration, with about twelve cells lying within the overlap of a given dendritic field, and the axonal-field overlap being 200–300 cells [45]. Thus even though the axon branches from any one PA1 cell are relatively sparse, the overall mesh of the resulting axonal plexus can almost be described as the woof and weave of a fabric [37], [45].

It should be noted that GABA may be the neurotransmitter in PA1 cells [37], [45]. One might think that this requires any spreading wave of spikes to be inhibitory, but there are numerous examples of positive communication and control links that have inhibition as a central component. This may be especially true where gap junctions are used in combination with inhibitory transmitters [48]. One could imagine, for example, that the electrical excitation being conveyed at a gap junction might be immediately terminated by chemical inhibition at that same locus. Thus activation of PA1 cells might result in ultrabrief signals that spread like rings through the axon branches, providing a physiological substrate for registering submillisecond coincidence of discrete stimulus events.

An explicit hypothesis about the role of PA1 cells has been suggested, but it should be said that there are numerous alternatives. Masland [49] estimates that there are upwards of 29 types of amacrine cells, all of which have extended processes that provide for lateral coordination of stimulus events. Those having spiking axons seem especially suitable for linking the activities of neighboring populations, which is one reason that PA1 cells were singled out for discussion. However, given the diverse and unexpected neuronal interactions that have been found in recent years, for example the ability of dendrite-to-dendrite linkage to code for local motion [50], it would be wise to watch for other possibilities.

A final question that should be addressed is why temporal proximity should be a major factor in the effectiveness of the shape cues. From an evolutionary perspective, developing special filters that register simultaneity might provide a way to identifying objects that are moving behind heavy occlusion, such as dense vegetation. Simultaneous changes in luminance across a myriad of openings in the vegetation might well provide the only cue to the existence of the object, and recognition of its shape would hinge on being able to piece together the pattern of these events. Further, encoding of images for purposes of storing and retrieving shape memories may well involve sampling over brief intervals, and if so, then the need to coordinate the sampling process could place a premium on the simultaneous presence of stimulus components.

Wertheimer [51] used the term “common fate” to describe the ability to see the gestalt of a moving pattern of points. While we commonly think of gestalt operations as being very cognitive, the early advocates of these concepts believed them to be fundamental, as reflected in the Kohler [1] quote given at the outset. The current evidence supports Kohler's conjecture that the process begins in the retina.

## Methods

Recognition judgments were collected from 33 participants, 12 each for the first two experiments, and 9 for the third. Participants were asked to name objects that were displayed using a minimal transient discrete cue (MTDC) protocol that has been detailed previously [2]–[6]. One hundred fifty shape patterns were shown to each participant, each being displayed only once.

As implemented in the present work, the MTDC protocol presented sparse sets of dots that marked the outer boundary of each shape, these being designed as “display sets.” The display set for a given shape was slightly different for each subject. It was chosen from the full inventory of dot positions that marked the outer boundary of the shape, beginning at a starting position and then sampling every Nth dot (see Figure S1). The value of N, designated as the spacing factor, determined the number of dots in the display set. The size of the spacing factor was chosen with the goal of having recognition level in the 75% range when all dots in the set were shown with no temporal separation (see timing specifications, below). Some reduction from this level of recognition was expected as a function of the spatial separation of pairs in the each of the experiments [6]. The names of the shapes, and the size of the display sets can be found in Table S1.

The dots of a given display set were shown as successive brief flashes within a 64×64 LED array, designated as the display board. Participants viewed this display from a distance of 3.5 m. At this distance the diameter of each LED, specified as a visual angle, was 4.9 minutes, center-to-center spacing of LEDs was 7.4 minutes, and the dimensions of the full array, width and height, was 7.7 degrees. Each LED emitted at a wavelength of 660 nm, with a rise/fall time that was less than 100 ns, and with a luminance of 10 Cd/m^2^. LED luminance was measured using a Quantum Instruments LX photometer, with LED adaptor.

Room illumination was provided by occluded standard fluorescent light fixtures, brightness level being 13.3 lux, as measured with a calibrated Tektronix J 1811 photometer. Brightness of ambient illumination and LED emission were the same as for the “dim” condition of prior reports [3]–[6].

For each experiment, each dot in the display set was flashed for 0.1 ms, designated as T1. T2 specified the temporal separation of pair members, measured from offset of the first member till onset of the second member. T3 specified the temporal separation of pairs, measured from offset of one pair till onset of the next. For Experiment 1 the T2 interval for display of each shape was 0, and T3 was either 0, 2, 4 or 6 ms. For Experiment 2 the T3 interval was 3 ms for display of each shape, and the T2 interval was either 0.0, 0.5, 1.0 or 1.5 ms. For Experiment 3 T2 was 0 ms, and T3 was 0, 0.5, 1.0, 1.5, 2, 4, 6 or 8 ms. These timing conditions are illustrated in Figure S2.

In each experiment the display set was further broken into subsets consisting of dot pairs. For Experiment 1, pair members were chosen so that the axis of alignment for the pair was either predominantly vertical or horizontal. Shapes were assigned to one of the two alignment conditions, *i.e.*, vertical or horizontal, and also to one of the four T3 levels, providing a given subject with either 18 or 19 shapes for each of the treatment combinations. The shapes were chosen at random for display, which thus randomized the order of the treatment combinations.

Experiment 2 used only horizontal alignment of pairs. Shapes were assigned to one of the four T2 conditions, providing each subject with either 37 or 38 shapes for each treatment level. The order of shape display (and thus T2 level) was chosen at random.

Experiment 3 randomly chose which dots would be designated as a pair. Shapes were assigned to one of the eight levels of T3 interval, providing either 18 or 19 shapes for a given T3 level for each subject.

For each experiment the shapes were shown successively, allowing ample time for the subject to provide a name that was judged to be correct or not. Most participants answered in 2–3 seconds for any given shape. The experimenter was not aware of the alignment or timing condition that was being used for display of the shape.

A given shape was either correctly identified or not, which is a binary alternative. The resulting data are best evaluated using a generalized linear mixed model (GLMM) that treats the errors using a logit link function [52]. For this one calculates logit values, these being log _e_ (proportion/1−proportion). Treatment differences were compared using the standard error of the difference of these values. For each of the experiments, shape and subject were treated as random effects, and pair alignment, T2 or T3, when serving as treatment variables, were fixed effects. In Experiment 3 display set size (Dot#) was evaluated as a variate.

Figure plots of percent recognition were based on GLMM predictions, which were extremely close to the raw data means.

## Supporting Information

Table S1 Names of shapes and descriptive data The table names each of the 150 shapes that were shown to each subject, and gives the length of the perimeter and area (each specified as a dot count). The spacing factor specifies the sampling interval for forming the display set, as further explicated in Supplementary Figure A. This process determines the size of the display set (Dot#), which can also be specified as a percentage of the total number of dots in the perimeter (Dot%).
(1.48 MB EPS)

Figure S1 Method for selecting the display set The full array of boundary dots for a typical shape is shown. From the full inventory of dots, a display set was chosen for presentation to a given subject, this being an evenly spaced sampling from the full inventory of dots. The particular sample was selected by choosing a random starting point, shown here by the arrow, and then proceeding clockwise around the perimeter of the shape, choosing every Nth dot to be included in the display set. The value of N (the spacing factor) was varied to provide approximate equivalence of potential for recognition across the inventory of shapes. The display set is shown using filled dots, which have been increased in size for purposes of illustration. Unfilled dots would not be part of the display.
(0.74 MB EPS)

Figure S2 Timing the display of dot pairs For both experiments, dots were shown successively with a duration of 0.1 ms (T1). In Experiment 1, the second member of the pair was shown immediately upon offset of the first pair-member, i.e., T2 = 0, and temporal separation of the pairs (T3) was varied across durations that ranged from 0 to 6 ms. In Experiment 2, the T3 interval was a constant 3 ms for display of each shape, and T2 was varied from 0.0 to 1.5 ms. In Experiment 3, T2 was 0 and T3 intervals ranged from 0–8 ms.
(0.71 MB EPS)
